# Complete genome sequence of iron-oxidizing *Stutzerimonas stutzeri* strain FeN3W isolated from Catalina Harbor sediment

**DOI:** 10.1128/mra.00030-24

**Published:** 2024-05-03

**Authors:** Jin-Sang Yu, Annette R. Rowe, Joshua D. Sackett

**Affiliations:** 1 Department of Biological Sciences, University of Cincinnati, Cincinnati, Ohio, USA; California State University San Marcos, San Marcos, California, USA

**Keywords:** marine microbiology, lithotrophy, extracellular electron uptake, electromicrobiology

## Abstract

*Stutzerimonas stutzeri* strain FeN3W is an iron-oxidizing bacterium isolated from marine sediment. FeN3W’s 5.9 Mb genome encodes complete pathways for glycolysis, gluconeogenesis, TCA cycle, pentose phosphate pathway, and aerobic and anaerobic (nitrate) respiration. The genome contains 32 putative heme-binding proteins predicted to localize to the cell envelope.

## ANNOUNCEMENT

Microbial mineral oxidation is an important biogeochemical process in marine sediment ecosystems (1–3); however, the metabolic processes conferring the capacity for extracellular electron uptake from solid-phase minerals remain poorly characterized (4, 5). We report the complete genome sequence of *Stutzerimonas stutzeri* strain FeN3W, an elemental iron and poised cathode-oxidizing bacterium (2).

Coastal sediments were collected via 30 cm push cores from Catalina Harbor, CA (33° 25.23′ N, 118° 19.42′ W) in February 2013. FeN3W was electrochemically enriched from homogenized sediment cores and subsequently isolated on solid minimal marine media supplemented with elemental iron and sodium nitrate, as previously described (2). FeN3W was grown from glycerol stock in LBS+Ions broth (25 g/L Miller LB, 10 g/L NaCl, 3 g/L MgCl2•6H_2_O, and 0.15 g/L CaCl_2_•2H_2_O) overnight at 30°C and 200 RPM. DNA was extracted from a cell pellet with the DNeasy PowerSoil DNA Isolation Kit (Qiagen, Germantown, MD) and that sole extract was used for both Illumina and Nanopore sequencing. Briefly, Illumina libraries were prepared with the Illumina DNA Prep Kit, barcoded with 10 bp UDI indices, and sequenced on an Illumina NovaSeq (2 × 151 bp sequencing) at SeqCenter LLC (Pittsburgh, PA). Demultiplexing, quality control, and adapter trimming were performed with bcl-convert 4.0.3 (6). Long-read sequencing libraries were prepared with the Native Barcoding 24 V14 Kit (ONT, Oxford, UK) without shearing or size selection and sequenced using a R10.4.1 flow cell (FLO-MIN114) under high accuracy mode (280 bp/s) with a MinION sequencing device in-house. Basecalling, adaptor trimming, and default quality filtering were conducted with Guppy 6.4.6 (7). Illumina reads were not filtered due to an average Phred score of 36 per read as determined by fastQC 0.11.5 (8). Long reads were filtered using Filtlong 0.2.1 (9), removing reads <5,000 bp and the lowest quality reads comprising 10% of all sequenced bases. Long-read sequences were assembled *de novo* using Flye 2.9.1 (10). Illumina reads were aligned to the assembly using Burrows-Wheeler Aligner 0.7 (11) and polished using Pilon 1.24 (12) with the parameter—fix all for three rounds total. Assembly quality was determined using CheckM 1.0.18 (13) and QUAST 4.4 (14). The assembly was annotated with NCBI’s PGAP 6.6 (15). Taxonomy was determined using GTDB-tk 2.2.5 (16). Potential metabolic pathways were identified using KEGG’s GhostKOALA tool 2.0 (17). Proteins containing putative heme-binding motifs (CX_2–4_CH) were identified via Prosite (18, 19), and their localizations were predicted with PSORTb 3.0.3 (20). Default parameters were used unless otherwise noted. Computing resources were supplied and maintained by the Ohio Supercomputer Center (21) and the DOE Systems Biology Knowledgebase (22).

The FeN3W genome is comprised of a 4.99 Mb chromosome and 0.91 Mb plasmid, with a G+C content of 60.41% (Table 1). Flye 2.9.1 (10) indicated both assemblies were circular and nonrepetitive. GTDB (16) identified the nearest neighbor as *S. stutzeri* NF13 (GCF_000341615.1, ANI = 99.41%). FeN3W’s genome encodes complete pathways for glycolysis, gluconeogenesis, pentose phosphate pathway, TCA cycle, aerobic respiration, and dissimilatory nitrate reduction to ammonia or nitrogen gas. Furthermore, Prosite (18) identified 85 proteins with putative heme-binding motifs, of which 32 are predicted to localize to the cell envelope.

**TABLE 1:** Sequencing statistics for *S. stutzeri* strain FeN3W

Illumina raw reads accession no.	SRR23873165
Nanopore raw reads accession no.	SRR23873160
GenBank accession no.	CP136010 –CP136011
Assembly N_50_ (bp)^*a*^	4,997,791
Nanopore N_50_ (bp)	13,223
Nanopore total read length (bp)	714,644,844
Illumina total read length (bp)	643,017,490
Illumina paired-end read count	2,152,644
Nanopore read count	100,000
G+C content (%)^*a*^	60.41
Estimated genome completeness (%)^*b*^	99.88
Estimated genome contamination (%)^*b*^	7.7
Estimated Nanopore coverage (x)	121
Estimated Illumina coverage (x)	99
No. of contigs	2
Chromosome length (bp)	4,997,791
Plasmid length (bp)	909,734
No. of protein-encoding genes	5,429
No. of tRNAs	115
No. of rRNAs (5S, 16S, 23S)	4, 4, 4

^*a*^ Determined via QUAST 4.4 (14).

^*b*^ Determined via CheckM v1.0.18 (13).

## Data Availability

The raw Illumina and Nanopore sequencing reads have been deposited in the Sequence Read Archive and are available under accession numbers SRR23873165 and SRR23873160, respectively. The complete genome sequence with annotations has been deposited in GenBank under accession numbers CP136010–CP136011.
